# Integrating local indigenous knowledge to enhance risk reduction and adaptation strategies to drought and climate variability: The plight of smallholder farmers in Chirumhanzu district, Zimbabwe

**DOI:** 10.4102/jamba.v12i1.924

**Published:** 2020-12-15

**Authors:** Mashoko S. Grey, Current Masunungure, Amanda Manyani

**Affiliations:** 1 Department of Research, CSR Group Africa (Consultancy) Pvt Ltd, Harare, Zimbabwe; 2 Sustainability Research Unit, Faculty of Science, Nelson Mandela University, Port Elizabeth, South Africa; 3 Centre for Complex Systems in Transition, Faculty of Economics and Management Sciences, Stellenbosch University, Stellenbosch, South Africa

**Keywords:** adaptation, climate risks, climate variability, drought risk reduction, indigenous knowledge systems, livelihoods, Zimbabwe

## Abstract

This article focuses on drought risk reduction and climate change adaptation strategies adopted by rural households to sustain their livelihood activities. The overall objective was to understand the local household’s responses to the changing climate especially drought. The study was carried out in Chirumhanzu district in Zimbabwe and used a mixed methods approach combining 217 household surveys, targeted focus group discussions, participatory learning actions methods, key informant interviews and a document review. Household data were analysed using Statistical Package for Social Sciences and thematic content analysis was used for the qualitative data. We found that the majority of households showed awareness of several risk reduction and adaptation strategies to implement during and/or when drought was predicted, with 56% of the respondents stating stocking of grain as initial strategy. Other strategies adopted at household level included early planting (at first rains), conservation farming, planting small grains and dry planting. Indigenous and traditional knowledge systems and practices, including local people’s holistic view of the community and environment, were a major resource for adapting to climate change and drought risks. However, these indigenous knowledge systems and practices had not been consistently used in the existing adaptation and risk-reduction efforts. Indigenous knowledge was not sufficiently acknowledged and integrated into formal risk reduction and adaptation strategies, which resulted in limited success for external interventions. There is need for integration of local and indigenous knowledge systems and external interventions to build household livelihoods that are resilient to climate risks.

## Introduction

Two of the main challenges facing communities and governments in the Global South are the risk reductions related to hydro-meteorological hazards (disaster risk reduction, DRR) and adaptation to climate change (climate change adaptation [CCA]; England et al. 2016; Peek 2016:8). Climate change has implications with regard to hydro-meteorological DRR as it increases and modulates the underlying risk factors such as droughts (United Nations International Strategy for Disaster Reduction [UNISDR] 2015). Changing and varying climates often lead to changes in the frequency, intensity, spatial extension, duration and timing of extreme weather events and hydro-meteorological hazards (Intergovernmental Panel on Climate Change [IPCC] 2012, 2014). Drought hazards are extreme weather events of concern, whose frequency and intensity have been significantly increasing (Chagutah 2010; IPCC 2012, 2014; Mubaya & Mafongoya 2016:1–27; Mubaya et al. 2012:9–17). This increase in the frequency of hazardous episodes creates a new and exacerbating environment for potential disasters. Poor households and communities are therefore more vulnerable to droughts and other hydro-meteorological hazards because of their dependence on rain-fed livelihood activities, limited livelihood and risk reduction options, and low adaptive capacity (Nangombe 2013; Shamano 2010). Droughts are particularly significant hazards in Zimbabwe and Africa at large, accounting for approximately 25% of all natural hazards on the continent for the period starting from the 1970s to date (Serrao-Neuman et al. 2015:46–61).

Hydro-meteorological DRR activities and CCA approaches to deal with climate change need to be linked and coordinated as emphasised by both the UNISDR and the IPCC reports (IPCC 2014; Mercer 2010:247-261; Rivera, Tehler & Wamsler 2015:445-456; Turnbull et al. 2013). This emanates from the fact that the negative impacts of climate variability and change in communities increase their disaster risk and erode adaptive capacity, thereby creating a platform for future disasters. The vulnerability analysis approach, initially developed for poverty and food security studies, has become a unifying framework for the CCA and DRR communities. The 2014 IPCC SREX report identified the link between climate change and extreme weather events and what it meant for DRR and CCA in the context of sustainable development (SD). Institute of Global Environmental Studies (IGES 2016) further indicated that the world has arrived at a major turning point for the inception of three-world frameworks: SD, DRR and CCA coordinated responses to maximise on national and local level adaptation planning and implementation. Given this scenario, the disaster risk community encourages the use of tools, methods and policies that enhance the reduction of vulnerability to climate variability and change (Birkman & Teichman 2010:1–15; Gaillard & Mercer 2012:93–114; Schipper et al. 2015). The fact that the communities in Zimbabwe and Africa at large have survived till today with a growing population is an indication that they have developed indigenous mechanisms and strategies for DRR (Cuaton & Su 2020).

### Conceptual framing: Local indigenous knowledge, disaster risk reduction and climate change adaptation nexus

Droughts are not a recent phenomenon but their frequency of occurrence has recently increased. This suggests that since droughts have been in existence from time immemorial, and forefathers used methods that were relevant to their context to protect their livelihood activities. Some of these strategies are still applicable and are currently used by some households for reducing risk and adapting to drought and climate variability, whilst some indigenous strategies might be difficult to implement in the current context. However, utilisation and adoption of local practices and indigenous knowledge have the potential to improve drought risk reduction and adaptation to climate change and variability for rural households in the developing economies (Hiwasaki et al. 2014:15–27). Indigenous knowledge fosters the utilisation of available local resources that are within the reach of many households and hence better positioned to assist them (households) when facing the impacts of extreme weather events (Nyamwanza 2014:23–33). Therefore, understanding local practices is pivotal to assessing how households utilise local knowledge and resources to reduce risk and cope with and adapt to frequent drought events (Theodory 2014). Similarly, Hiwasaki et al. (2014) argue that local knowledge can give smallholder farmers an opportunity to utilise local farming practices that might reduce the impacts of drought and related climate risks. Investigating such local and indigenous knowledge, and the strong social interrelations associated with it, helps to reveal the functional flexibility of local farming strategies and can provide a gauge of local adaptive capacity and ability to cope with climate risks especially drought. Further, incorporating local practices can lead to the development of effective mitigation and adaptation strategies that are cost-effective, participatory, sustainable and ultimately promote livelihood resilience that bears the heritage values of the community (Hiwasaki et al. 2014:15–27). Therefore, DRR and CCA should build on local practices, something that is often poorly addressed or even ignored by practitioners supporting risk reduction and adaptation interventions (eds. Janicot et al. 2015). As such, complementarities need to be sought between local and external interventions to enhance communities’ adaptive and coping capacity to climate risks (FAO 2014a).

Drought risk reduction and adaptation practices advocated by international development partners can potentially reduce vulnerability by changing the context in which shocks and stressors occur, or they can directly address outcomes (England et al. 2016). However, some practices prescribed and introduced by international development partners may negatively affect livelihood activities existent in the community because of incompatibility, thereby increasing vulnerability to climate risks. With the current trends of climate variability and successive droughts, some argue that integrated technological and scientific interventions offer the best option for strengthening livelihoods through improved agricultural productivity and building the capacity of households to diversify income revenues (Shiferaw et al. 2014:67–79). However, it is important to pay due respect to the existing traditional and cultural systems, institutions and structures, especially at the community level, when offering recommendations for agricultural production (FAO 2014b; Lade et al. 2017:5). An integrated approach, which is embedded in combing drought risk reduction, poverty reduction through SD and CCA approaches, is thus most likely to see households build resilient livelihoods and build back better when hard hit by drought disasters.

There is growing consensus that the recovery process for rural households, including livelihood activities and assets, is influenced by the interface between climate risk policies, institutions and dynamic pressures in that area, as explained in the pressure and release (PAR) model (Audouin et al. 2013:12; Santha 2018:65–78; Twigg 2015; Winser et al. 2004). Essentially, the PAR model shows that a disaster is an intersection between socio-economic pressure and physical exposure (Winser et al. 2004). In an empirical study in Zimbabwe, Nyamwanza (2014:23–33) shows that policy frameworks need to be formulated and, most importantly, interpreted and implemented within an understanding and acceptance of local realities, if rural households are to build adaptive capacity or recover from drought disasters. In this regard, the recovery process not only requires adequate time but also depends on the extent of adverse impacts of the previous disaster, referred to as unsafe conditions in the PAR model, and accessibility to available local resources and assets. Sallu et al. (2010:3–15) point out how many climate-induced disasters (including drought) can destroy or damage the natural resource base and climate-sensitive activities of a community. Hydro-meteorological disasters adversely affect future livelihood prospects over both the short- and the long-term. In such cases as evidenced from this study, household recovery becomes difficult if not impossible, as the households face challenges to reduce risk and adapt to climate risks, that is, drought and climate variability.

## Methodology

### Study area

**FIGURE 1:**
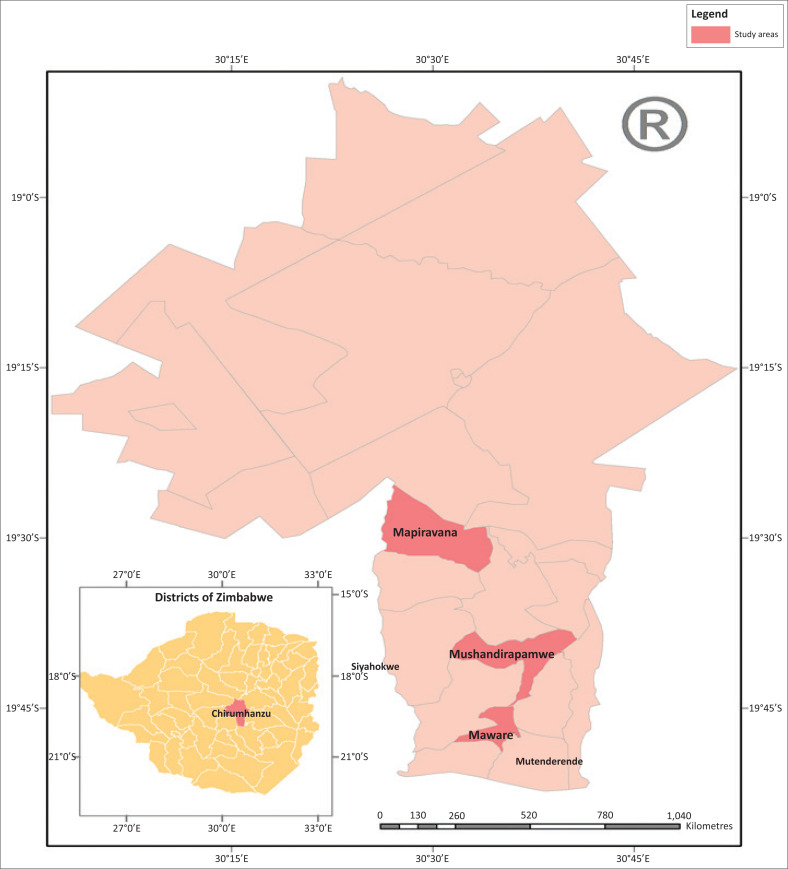
Map showing selected wards of Chirumhanzu district.

The study area is located in Chirumhanzu rural district (Midlands Province) in Zimbabwe, which is one of the eight districts in the Midlands province. Chirumhanzu district is divided into 25 administrative wards and the study was undertaken in Wards 1, 10 and 25 (Figure 1). Chirumhanzu district has a total of 19 736 households and a total population of 81 087 with 47% males and 53% females (Zimstat 2012). Chirumhanzu district lies in agro-ecological regions 3 and 4 where semi-intensive mixed farming and extensive farming with livestock ranching is suitable and recommended because of the aridity of the area. The district is located mainly in the mid-altitude areas of the country and is characterised by an annual rainfall of 500 mm – 750 mm, midseason dry spells and high temperatures (Mugandani et al. 2012:361–369). The district was therefore purposively selected, as it is one of the areas frequently affected by hydro-meteorological hazards and specifically droughts, over the years.

### Methods

This study used a mixed methods approach which allows for triangulation of quantitative and qualitative data and enhances the likelihood that data collected provided greater insights, depth and quality (Creswell & Plano Clark 2007; Denscombe 2008:270–283). Data were collected through document review, key informant interviews (i.e. Agricultural Extension Officers, Climate Change Management Department Officers, Department of Civil Protection), household surveys, focus group discussions (FGDs) and transect walks. Household survey questionnaires were administered to 217 respondents^1^ across three randomly selected wards: Mapiravana (Ward 1), Mushandirapamwe (Ward 25) and Maware (Ward 10).

Household survey questionnaires were administered to 217 respondents across three randomly selected wards. The sample size selection for the households assumed that 25% of the rural population has been affected by drought, with a desired 95% confidence interval and precision of 0.05% as indicated in the formula below:

$$n=t^{2}\times p(1-p)=1.645^{2}\times 0.25(1-0.25){\text{m}}^{2}0.05^{2}=203$$ (Eqn 1)

where *n* = sample size and *t* = confidence level at 95% level of significance (1.96).

Based on the above calculation, the resultant sample size for the total households interviewed for three wards was, therefore, 203 × 1.5 × 0.05 = 217 households.

A total of six FGDs (two FGDs per ward) were held to elicit ideas, insights and experiences in a social context, where people were stimulated to give their own views (Mubaya et al. 2012). Three FGDs had only males participating and three FGDs had only females participating, that is, two FGDs per ward across three wards. These FGDs were conducted separately to ensure that women’s voices were not marginalised or to ensure that they could speak openly and freely without any power dynamics coming into play. Ten key informant interviews (KIIs) were conducted at the district and national levels mainly to get policy views and technical information not attainable at the household level. Transect walks with three volunteers from the community provided an opportunity to explore the current agricultural practices and other livelihood strategies for different households.

As the study collected both qualitative and quantitative data, the data analysis methods used matched the nature of the data as indicated below. Qualitative data were collected as interview scripts and observational notes from key informant interviews, transect walks and FGDs. The interview and discussion recordings were transcribed from Shona to English for analysis. For this study, thematic qualitative data analysis was employed in the rigorous ordering and structuring of the qualitative data. Thematic data analysis is the method of ‘identifying, analysing, and reporting patterns (themes) within data’, and it is also a descriptive method that reduces the data in a flexible way that dovetails with other data analysis methods (Mubaya et al. 2012). The themes were then linked together into chains or patterns of evidence to enable the drawing up of contrasts and comparisons in the experiences of the community. The Statistical Package for Social Sciences (SPSS) Software Version 20 was used to analyse quantitative data collected from household surveys. Responses to the household survey questions were coded for entry into the SPSS software after the data collection process. The SPSS file was used to generate frequency tables, percentages of responses and cross tabulations. Overall, the analysed data were presented using tables, graphs and charts.

### Ethical consideration

The verbal permission to collect data was granted by Provincial Administrator for Midlands Province and the District Administrator for Chirumhanzu District.

## Findings

### Drought risk reduction and adaptation strategies for crop production

**FIGURE 2:**
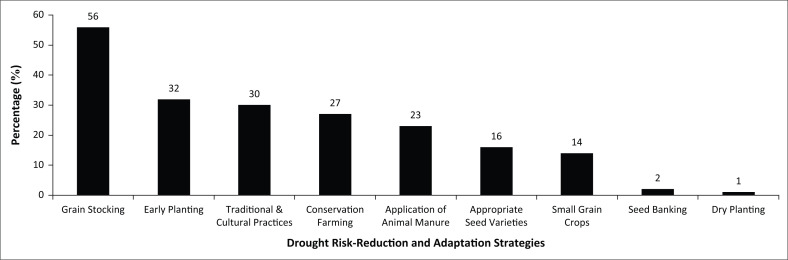
Drought risk reduction and adaptation strategies (multiple responses, *n* = 217).

Regarding arable cropping, several strategies, both endogenous and introduced, were used by the interviewed smallholder farmers to deal with drought or expected drought. Relevant government departments and NGOs issue advice using local structures and mass media (radio, television and newspapers) to try and reach out to the majority of rural households. Seventy per cent of the smallholder farmers knew what strategies to adopt whilst 30% did not, when facing or experiencing drought hazard. From those smallholder farmers who knew the strategies to use, 56% stocked grain (largely maize) until the next harvest season (Figure 2). The grain was mainly stocked in grain store structures that were built using locally available materials, that is, roof with thatching grass, timber, clay bricks and stones for foundation. Focus group discussion participants in all wards supported this and mentioned that stocking of grain until the next harvest was their primary and initial step to ensure continued food availability during lean periods at household level:

‘… we even reduced the size and number of meals taken per day to one meal per day in the evening, porridge in the morning to save on mealie meal and in the afternoon everyone would fend for themselves with fruits or food from neighbours and friends …’ (Female, FGD Participant, Ward 1)

However, transect walk participants in all wards revealed that some household granaries did not portray features that guarantee security and proper grain storage conditions. This is largely because households could not afford to purchase building materials for construction of proper granaries as recommended by Agricultural Extension Officers for post-harvest grain management (Figure 2).

Using observed indicators or relationship with events, for example first rains, one-third of respondents planted early with the first rains of the new cropping season in selected areas. The measures of reliability were based on households’ experiences of failure of these indicators:

‘… the practice of early planting of crops was largely applied in dambo^2^ fields (“Doro” in local name), which are now less prevalent because of the change in the amount of rainfall received annually.’ (Male, FGD Participant, Ward 1)

Smallholder farmers with *dambo* fields planted their maize crops as early as September and harvested in December, often planting another crop (i.e., round nuts, groundnuts and finger millet) from December until April. With this strategy, households increased the chances of their crops receiving as much moisture as possible and increased their chances of a decent harvest. However, some households indicated some difficulties because of (1) the limited rains received annually that now largely affect these *dambo* fields and adversely affect their water-holding capacity and functionality and (2) long midseason dry spells and unpredictability of rains made it difficult for households to prepare for the early season. Reduced rainfall amounts because of climate variability and change dries the *dambo* fields; hence, smallholder farmers will not be able to practice early planting, a strategy for drought risk reduction. With reduced rains, *dambo* fields cannot retain their water holding capacity and cannot be wet even during the dry season of the year and hence loose their functionality. Another one-third engaged in traditional and cultural practices to influence the weather, especially rainfall (rituals), whilst 27% used conservation agriculture (Figure 2). The main traditional practice performed was a rainmaking ritual known as ‘mutoro’ or summoning the rains. However, other traditional and cultural practices such as the ‘zunderamambo’ (contributing grain to the chiefs’ granary, which will be used during lean periods) are no longer practised, exposing vulnerable households to the vagaries of successive droughts.

Another proportion of respondents adopted the traditional application of livestock manure to the fields to improve soil fertility (23%), 16% used appropriate seed varieties (hybrid seeds tolerant to drought), 14% grew small grain crops whilst 2% used seed banking (Figure 2). Only 1% of respondents used dry planting^3^ (Figure 2). In relation to seed banking, study participants revealed that the selected maize cobs were smoked in a traditional rural kitchen mainly as a treatment against pests and insects (weevils and borers). Using, for example, the state of flora and fauna and constellation of stars, smallholder farmers would know when to harvest the crop to ensure that grain had appropriate moisture content and received the required treatment to preserve it for a long time. However, in other cases, households used harvested grain for planting without special selection from the grain.

Some respondents believed that performing the traditional rituals ensured good rains and consequently a good harvest. Some FGD participants also mentioned that rainmaking ceremonies were effective in the past to the extent that it would start raining immediately after the ritual:

‘… in the past, upon completion of “mutoro” ritual, the rain would come indeed. Nowadays the rain seems to even go into hiding when we perform the rituals. Some among us attend churches and we go to the fields with the priests to pray for the rain. Either way, the rains are asked for in October. No rituals or ceremonies are performed in November. Anything performed after October is futile because the rains have started already. The effectiveness of “mutoro” is arguable because the processes are not being done correctly. Some of the information on conduct of traditional rituals seems to be lost in translation. The knowledge is still there with the elders and not passed on to the younger generation. There is also desecration of sacred areas, which could be leading to erratic rains …’ (Male, FGD Participant, Ward 10)

However, other FGD participants also stated that traditional rainmaking rituals were currently not working at all mainly because the rituals were not conducted in the appropriate manner:

‘… we had “mutoro” last week in one of our villages but unfortunately it did not even rain after the ceremony. It seems misleading these days. In some cases, it seems to scare the rains away. It may be due to the fact that the elders are not passing on the information accurately or the young are not interested. We try to instruct the youth on culture, but the young ones who are recently married are arrogant and adamant, saying that the water is from God …’ (Male, FGD Participant, Ward 25)

Focus group discussion participants expressed different views on introduction of conservation farming in their communities. Some participants indicated that conservation farming was introduced to help households without draught power for ploughing, whilst others, specifically from Mapiravana ward, argued that conservation farming was introduced by NGOs together with the provision of farming inputs to incentivise smallholder farmers to embrace the practice, given the occurrence of successive droughts. Overall, it was revealed that conservation farming required intensive labour, which was too much for the elderly and small households and hence was used mainly on small pieces of arable land:

‘… I am happy with the introduction of conservation farming in my area as I am guaranteed good harvests every season. I know it is labour intensive, that is why I use it for my homestead field only, and this is the only portion I cultivate. As you can see, I am going to have a bumper harvest this season 2014/15 when the majority of the population in my area will be suffering from poor harvest. The problem with many people in my area is that they do not want to work hard, they are just lazy and they do not want to use conservation farming. Conservation farming works and it works for me …’ (Female, FGD Participant, Ward 25)

Smallholder farmers who used conservation farming indicated that it helped them to realise good harvests throughout the past drought years.^4^ However, most of the smallholder farmers especially in Mapiravana ward had stopped using conservation farming when the NGOs that had introduced it departed, as there was no longer any incentive in the form of inputs and also that it is labour intensive.

The agricultural extension officers under the Ministry of Lands, Agriculture, Water, Climate and Rural Resettlement provided advice to smallholder farmers on the type of seed varieties suitable for each season depending on the seasonal weather forecasts. Overall, the agricultural extension officers recommended that in the face of drought, smallholder farmers use drought resistant crops and seed varieties and early**-**maturity maize seed varieties to stand a better chance of attaining better yields. In this regard, early**-**maturity varieties^5^ were recommended as they fit into the shortened rain season. Agricultural seed companies in Zimbabwe, such as Pioneer, Pannar and SeedCo, have been developing hybrid varieties that are drought resistant and disease tolerant, and produce high yields. In turn, observations in hardware shops in the study site showed that different types of hybrid seed varieties were now being sold to the surrounding communities. However, many smallholder farmers in the FGDs could not afford to buy treated hybrid seeds every cropping season because of limited financial resources. In those cases, households used traditional untreated seeds from previous harvests or seeds facilitated by seed banking. However, one FGD participant from Mushandirapamwe ward pointed out:

‘… the yield from untreated seed would not be impressive even with adequate rains in comparison to hybrid seeds …’ (Male, FGD Participant, Ward 25)

With regard to livestock, results indicated that the main concerns for livestock when struck by a drought disaster were water and fodder. The majority of respondents (87%) kept cattle feed (maize stalks) to ensure the survival of livestock during droughts. Other options were to fetch water for livestock (9%) and to travel long distances in search of water and pasture (4%). The latter were important considering that many water sources in the study site dried up during droughts. With regard to feeding livestock, the FGD participants from all wards also indicated that during drought, livestock were herded along the river banks that usually remained green and provided a source of fodder.

### Households’ perceptions on recovery after drought disaster

In this study, recovering from drought disasters was based on households’ perceptions of retaining to their status quo or better, before the next drought. Households perceived that droughts were now frequent to the extent that recovery before the next season was vital to ensure that impacts of the next drought were reduced or contained. With regard to households’ recovery after drought, 38% of respondents mentioned that they were able to recover, 34% said they did not recover timeously before the next drought and 28% mentioned that they never recovered at all. Thus, a combined two-thirds of respondents struggled to recover from drought impacts, which increased vulnerability and resulted in drought disasters despite external interventions by development partners. External support includes (1) the intervention provided food during difficult times, (2) food aid was often people’s only source of food during drought events and (3) farmers received training on farming techniques (Christian Care & Oxfam International). Overall, in the KIIs and FGDs, it was highlighted that droughts could stretch for more than one season and the food situation, especially in the later seasons, could become dire resulting in increased demand for food aid. The support received during the past drought years was in the form of food handouts, especially maize grain, sugar beans, cooking oil and barley to prevent starvation and malnutrition; and these were largely received from the World Food Programme and Oxfam International.

## Discussions

### Smallholder farmers and crop productivity during drought

Because food shortage is one of the greatest impacts of drought, conserving grain stocks until the next harvest was an important strategy for households in Chirumhanzu district (Bola et al. 2013:180–186; Connolly-Boutin & Smit 2016:385–399; Nhemachena et al. 2014:123; Rankoana 2016:672). Households in this study considered grain stocking as an initial step towards reducing drought impacts. An important economic benefit of safe grain storage is that smallholder farmers affected by drought are not under pressure to sell their grain (FAO 2014b). This would increase their bargaining power, as they have an option to delay selling their grain whilst seeking better prices (FAO 2014a). Failure to stock grain has been shown to undermine the capacity of rural households to overcome drought crises and impede on early recovery after drought events (FAO 2014a). In an empirical study in Zimbabwe, Mawere, Madzima and Mabeza (2013:14–23) also showed that the lack of grain reserves during drought years already pushes households into the one meal per day bracket. However, storing grain for a long period without damage requires quality storage structures; yet granaries built by some households (Mushandirapamwe ward) were not in accordance with the standards as advised by agricultural extension officers. Quality storage structures are crucial in keeping out pests and insects as well as maintaining dry conditions. The lack of such structures in some households limited the ability of households to stock for longer periods and then sell grains during drought periods, when they could get higher returns because of high grain prices. This resulted in limited income to meet other household demands.

In this study, conservation farming was largely introduced through NGOs (e.g. Christian Care and Oxfam) and agricultural extension officers to assist smallholder farmers during dry seasons. Households that used conservation farming clearly indicated their satisfaction that conservation farming helped them to realise good harvests from their small fields (homestead fields) during drought episodes. Sietz and Van Dijk (2015:131–141) argue that larger families with a large proportion of active members are more likely to use conservation farming on a large scale as it is labour intensive. Small families that still used conservation farming would reduce the size of their fields (under conservation farming) to reduce labour requirements. That said, if each household in rural areas was to set aside a small piece of land (at least 1 acre) for conservation farming and grow small grain crops, it would go a long way towards fostering food security in the district that is characterised by frequent droughts. However, this study’s findings indicated that growing of small grain crops by households has been slow, which contradicts the findings by Mawere and Mubaya (2015) who indicate that small grain crops are increasingly becoming dominant crops because of their drought resistance characteristics. The slow uptake of drought-tolerant crops could perhaps be attributed to several factors that included: maize is the nation’s staple crop and a shift from this crop might result in a change in household diet, which rural households are likely not prepared to do; limited information at the household level on the impact of drought on crops (especially maize) and the benefits of drought-tolerant crops (especially small grain crops). Small grain crops can foster households to cope with drought because of their drought tolerance and favourable long-term storage that can cushion households during successive drought periods. Smallholder farmers’ slowness to adopt drought-tolerant crops can only result in unsafe conditions that favour drought disaster occurrence, as explained earlier in the PAR model (Sietz & Van Dijk 2015:131–141; Winser et al. 2004).

Households that could afford to purchase farming inputs largely used appropriate drought-tolerant seed varieties for their farming activities. Some households benefitted from the government input scheme that provided seeds and fertiliser to communal smallholder farmers. However, even for those households that benefitted from the government input scheme, the inputs received were inadequate for the earmarked pieces of land for farming and inappropriate because of the seasonal forecasts and/or prevailing climate variability and change. This left smallholder farmers with no option other than to top-up their processed seeds with the traditional unprocessed seeds. Some hybrid seed varieties are drought and disease resistant and have a high probability of producing high yields during drought. Despite clear advantages of these hybrid seeds, especially with regard to drought tolerance and yield, they require costly inputs, such as fertilisers and pesticides, that may not be available or accessible financially to smallholder farmers to obtain their maximum yield potential (FAO 2014a, 2014b). However, it is argued that using hybrid and drought-tolerant seeds will promote adaptive capacity to farming activities (McGuire & Sperling 2016:179–195). Yet, there were two arguments concerning traditional seeds as revealed in the study. Firstly, traditional seeds have limitations that include lower yield potential and substandard quality (irregular viability and reduced physical and varietal purity), leading to weaker yields over time (FAO 2014a). Secondly, smallholder farmers prefer traditional seed varieties because they are cheap and accessible and are perceived to be better adapted to withstand stresses such as droughts (FAO 2014b). This perception creates conflicts amongst the smallholder farmers and results in many of them being adversely affected by drought as traditional seeds cannot withstand droughts and/or inconsistent rains. Therefore, the inability of the majority of smallholder farmers to move to treated hybrid and drought-resistant seeds or treat their own traditional seeds could result in poor harvests becoming imminent and consequently food insecurity requiring. This also results in food aid through development agencies becoming a regular requirement in these communities (Bola et al. 2013:180–186).

### Livestock and droughts

Harvested and stocked maize stalks were one of the most important sources of cattle feed during the dry season and during drought periods when pastures were dry with insufficient fodder. The maize stalks were then fed to livestock in small portions until the beginning of the rainy season when new growth of grazing appear in the rangelands. However, keeping such feed for the livestock is dependent on the availability of maize stalks, which were also affected by drought disasters (Bola et al. 2013:180–186; Thornton, Boone & Ramirez-Villegas 2015). Further, maize stalks kept as livestock feed are unlikely to last the full length of the drought period, especially when hit by successive droughts. During severe droughts, the supply of maize stalks for livestock would be at best limited. With inadequate maize stalks for the entire drought period, smallholder farmers were forced to seek distant water sources and greener pastures especially along river banks. Fetching water for livestock was considered the last option after all other water sources had dried up in the community. This was done largely at boreholes constructed with small ponds for livestock. Depending on the severity of the drought, some households had to travel as far as 5 km. This meant that households with young and able-bodied members were prepared to travel long distances. With regard to pasture, the main concern was that the small pieces of green pasture along rivers were overwhelmed by livestock during drought periods.

### Smallholder farmer’s recovery from drought disasters

Households falling into the category ‘able to recover’ were likely endowed with a wide livelihood asset base, diversified livelihood strategies and various sources of off-farm-based income (all these contribute to adaptive capacity of the households) as explained by DFID (2011) resilience framework. Inversely, respondents in the category ‘never recovered at all’ were likely to have a shallow livelihood asset base and be heavily reliant on rain-fed subsistence farming with limited off-farm income. With droughts now occurring more frequently, almost every 2 years or even annually in some cases, many households are not able to recover because of the combination of low adaptive capacity and the frequency of extreme weather events. Recovery from drought disasters requires the rebuilding of livelihood assets, which depends on favourable structures and processes, as explained in the PAR model (DFID 2011; Sango & Godwell 2015:1–6). Poor households are unlikely to build back better during the recovery process, and this creates another vulnerable platform for future climate risks to escalate to disasters (Brown, Doldman & Zvigadza 2013). Therefore, they are likely to fall deeper into poverty as they face more frequent droughts with limited livelihood assets and livelihood options (Connolly-Boutin & Smit 2016:385–399). In a similar study in Zimbabwe, Rurinda et al. (2014:65–78) showed that many smallholder farmers who experienced the 1991–1992 drought and lost cattle have not yet recovered and will not be able to do so without sustained and meaningful external support. This failure to recover was attributed to, amongst other factors, the successive droughts, the ailing economy that limited households’ opportunities for income and the importance of livestock as a source of cash savings.

### Local and indigenous knowledge and practices

Some households believed that indigenous strategies could not be adopted into the current context and this was probably a reflection of socio-economic, political, environmental and demographic changes that had occurred over the years. For example, the chief’s common granary (*zunderamambo*) coping strategy might no longer be applicable to the current context because of the following perceptions: (1) weakened social cohesion in communities, (2) the introduction of formal administrative offices representing local government might be undermining the power of traditional leaders and other lower power structures, (3) modern ideologies and religion have turned most people from believing in traditional rituals and practices and (4) continuous poor harvests because of successive droughts. The powers and influence of the current chiefs have been diluted by administrative structures set up by rural district councils through the appointment of district administrators and ward councillors as well as ward development committees and village development committees. These structures compete with the traditional ones, making it difficult for the chiefs to enforce traditional and cultural practices.

## Conclusion

Different drought risk-reduction and adaptation practices for subsistence crop production and small-scale livestock rearing were used at the household level when drought was predicted and/or during drought events. Some households adopted practices based on local knowledge whilst others adopted practices advocated through development partners’ interventions and agricultural extension services. The following factors were behind households adopting local knowledge and practices: (1) they use local resources for cost-effective local solutions and (2) these local practices had been used before by their forebears. The success of these local and indigenous practices was also dependent on the severity and intensity of the drought and the nature of interventions (development partners) extended to households. With successive droughts, local and indigenous practices used by resource-poor smallholder farmers were often overwhelmed and not sufficient to build resilient livelihood strategies. Also, interventions through development partners in the form of food aid (e.g. maize grain, wheat grain, sugar beans and cooking oil) have been ongoing during these frequent drought periods creating a dependency syndrome amongst community members. Other households are now reluctant to engage in activities that can save their livelihood strategies knowing they would get food assistance from international development partners. In this article, we argue that there is a need to integrate local and indigenous knowledge and contextually analyse local indigenous practices on a broader scale considering the current changing climate and assess what might continue to be relevant and what can be strengthened by science for improved livelihood strategies (Nhemachena et al. 2014:123). Furthermore, poverty and development strategies should be mainstreamed in CCA planning, so that rural households livelihoods become resilient to successive climate risks (Lemos et al. 2013:437–457).

## Footnotes

1 The respondents for this study were the adult of each household, either the household head or the spouse. The households were sampled in a such manner that the households with no respondents were replaced with the nearest households, hence reaching the targeted sample size.

2 *Dambos* are seasonally waterlogged areas found in the headwater zones of the drainage systems or alongside streams or rivers (Turner 1986). Their importance spans from providing grazing in the dry season as well as being moist enough to grow dry season crops without irrigation (ibid.).

3 Dry planting is a practice whereby farmers plant the seeds a week or even 10 days before the onset of the rains. This practice ensures that as soon as the rains commence, the planted seeds will begin to germinate. The seeds must be planted in dry soil and the rains must come within that period or farmers risk their seeds rotting underground.

4 Conservation farming is a farming practice based on three principles: minimum soil disturbances, permanent soil covers and crop rotation. These are known to improve soil quality, conserve moisture and most of all improve yields during moisture stress periods (FAO 2008).

5 An early-maturity variety can either be planted early and harvested before the end of season, or be planted late and harvested by the end of season. It is recommended in areas where the rain season is short, rainfall patterns are irregular or in situations of chronic drought (FAO 2014b).
